# Vaginal Delivery at Term in a Woman with a Spontaneous Heterotopic Pregnancy Treated with Laparoscopic Salpingectomy

**DOI:** 10.1155/2020/8892273

**Published:** 2020-09-02

**Authors:** Michail Diakosavvas, Nikolaos Blontzos, Georgios Daskalakis, Athanasios Protopapas, Nikolaos Kathopoulis, Panagiotis Antsaklis, Grigorios Derdelis, Kyveli Angelou, Zacharias Fasoulakis, Dimitrios Loutradis, Marianna Theodora

**Affiliations:** ^1^1st Department of Obstetrics and Gynecology, Alexandra Hospital, National and Kapodistrian University of Athens, 80 Vasilissis Sofias Avenue, 11528 Athens, Greece; ^2^Department of Obstetrics and Gynecology, Mitera Hospital, 6 Erythrou Stavrou Street, 15123, Marousi, Athens, Greece

## Abstract

*Background*. The coexistence of an intrauterine pregnancy and an ectopic pregnancy (heterotopic pregnancy) is an extremely rare, yet major, complication during pregnancy. The early diagnosis of a heterotopic pregnancy is of great importance for fetal viability, maternal safety, and the progression of an uncomplicated intrauterine pregnancy. *Case Presentation*. We report a case of a naturally conceived heterotopic tubal pregnancy in a 37-year-old primigravida. The patient presented with continuous, dull, lower abdominal pain and a positive urine pregnancy test which was conducted a week prior to the start of the pain. The patient was hospitalized, and based on the clinical image and after strict monitoring, she was diagnosed with a heterotopic pregnancy. She was treated with laparoscopic salpingectomy after the rupture of the ectopic pregnancy while the desired intrauterine gestation continued without any complications. The pregnancy resulted in the birth of a healthy infant through vaginal delivery. *Discussion*. Strict monitoring with multiple sonographic evaluations should always be conducted in women with abnormal serum beta-hCG, adnexal abnormalities, or clinical symptoms, while heterotopic pregnancy should be in differential diagnosis and treatment should not be delayed since emerge management is important for the progression of the intrauterine pregnancy.

## 1. Introduction

The coexistence of an intrauterine gestation and an ectopic gestation is defined as heterotopic pregnancy, and it is a very rare condition with a frequency estimated to be less than 0.003% (1 in 30,000 cases) in natural conceived pregnancies [1]. Assisted Reproduction Technologies have increased the occurrence of coexisting intra- and extrauterine pregnancies, currently reported to be varied between 1 in 100 and 1 in 500 cycles [2–4]. Given that heterotopic pregnancy is considered a major complication of a pregnancy, the early diagnosis is of high importance for the therapeutic management of this condition and is directly associated with increased maternal and fetal morbidity and mortality [5, 6].

Herein, we present a rare case of a naturally conceived heterotopic tubal pregnancy in a 37-year-old nulliparous woman that was treated with laparoscopic salpingectomy after the rupture of the ectopic pregnancy that resulted in the birth of a healthy infant through vaginal delivery at term.

## 2. Case Presentation

A 37-year-old, nulliparous patient was admitted to our Emergency Department complaining about continuous, dull, lower abdominal pain. She had a history of a positive urine pregnancy test a week ago, with her last menstrual period indicating a pregnancy of 5 weeks and 5 days. Her past medical and surgical history was uneventful. She had not been subjected to any fertility treatment modalities and reported no risk factors connected to ectopic pregnancy.

**Figure 1:**
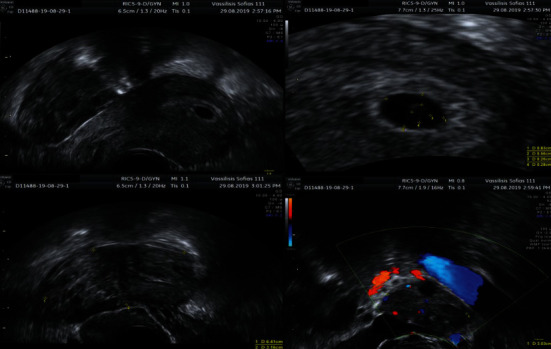
Hypoechoic mass in the right fallopian tube/adnexa with minimal blood flow on Doppler scan and illustration of the intrauterine pregnancy.

Clinical examination revealed a mild tenderness in deep palpation of the right lower quadrant without signs of peritoneal irritation. Pain was elicited during physical examination, located at the right adnexa, and no vaginal bleeding was recorded. The patient was hemodynamically stable. Her initial blood test results were within a normal range, except from an elevated serum beta-chorionic gonadotropin at 5436 mIU/mL. Transvaginal ultrasound was performed, and the findings suggested multiple, subserosal fibroids (with the greatest diameter: 41 mm), a single intrauterine gestational sac (mean diameter 7.3 mm), including a fetal pole with a crown-rump length of 1.7 mm and positive cardiac activity. In the right fallopian tube, a hypoechoic mass measuring 64∗32∗29 mm with minimal blood flow on the Doppler investigation was depicted, while the Douglass pouch was free of fluid. The ovaries appeared normal. Differential diagnosis included hydrosalpinx, hematosalpinx, ovarian torsion, and heterotopic pregnancy (Figure 1).

**Figure 2:**
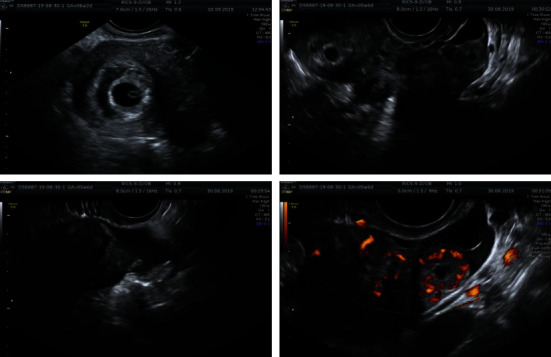
Simultaneous sonographic appearance of intrauterine and tubal ectopic pregnancy and free fluid in the Douglas pouch.

Due to persisting mild pain, the patient was hospitalized and monitored in our obstetrics department. The patient remained clinically stable and was subjected to additional transvaginal sonographic evaluation daily. On the 3^rd^ day of hospitalization, the ultrasonographic evaluation revealed a viable intrauterine pregnancy and mild accumulation of free fluid in the lower abdomen, suggesting a possible rupture of a coexisting tubal pregnancy. At that time, the patient complained about pelvic pain more acute than usual alongside with nausea while the vital signs and hemoglobin level were within the normal range (Figure 2).

**Figure 3:**
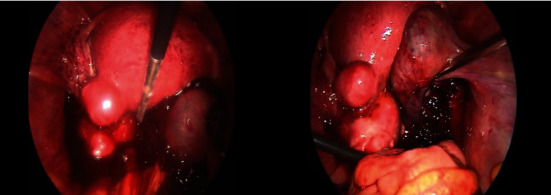
Hemoperitoneum. Ruptured ectopic pregnancy of the right fallopian tube.

After extended counselling and written consent, an emergent exploratory laparoscopy was performed. Under general anesthesia, with the patient in a lithotomy position, trocars were placed in standard sites, with intention to avoid any injury of the uterus, with the least possible manipulations, in order for the intrauterine pregnancy to be preserved. A mild amount of blood and clots and an ectopic pregnancy with active bleeding in the ampulla of the right fallopian tube were visualized, and right salpingectomy and evacuation of hemoperitoneum were conducted. A single intravenous dose of antibiotics was administered intraoperatively, and immediate confirmation of fetal heartbeat of the intrauterine gestation was done postoperatively (Figure 3).

**Figure 4:**
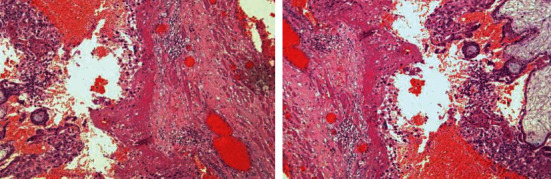
Implantation site with trophoblastic cells in the fallopian tube and chorionic villi in the lumen.

Oral progesterone was administered in a daily dose of 600 mg in order to prevent a miscarriage of the intrauterine pregnancy, due to possible lack of corpus luteum. After an uneventful recovery, the patient was discharged 2 days postoperatively with a viable ongoing pregnancy of 7 weeks. Recommendations concerning routine antenatal assessment were given. The final histopathological analysis of the specimen confirmed the diagnosis of an ectopic pregnancy. After an uncomplicated full-term pregnancy (40 w and 1 day), a healthy, female newborn weighting 3295 g was born via a spontaneous vaginal delivery (Figure 4).

## 3. Discussion

Until today, only a small number of naturally conceived heterotopic pregnancies have been reported, and even a smaller number of them have resulted in the delivery of a live-born infant [7]. The most common site of a heterotopic pregnancy is the fallopian tube, followed by the uterine cornua [5, 6].

The main risk factors of heterotopic pregnancy, reported in 70% of the patients, are fertility treatment techniques (such as ovulation induction or multiple embryo transfer), previous tubal surgical procedures (such as salpingotomy, salpingectomy, or tubal ligation), and tubal damage (such as hydrosalpinx and tubal adhesions as a result of pelvic inflammatory disease or endometriosis) [1–3, 6–9]. Nonetheless, as illustrated in our case, no predisposing factor was present, suggesting that other yet unknown types of tubal pathology may play a role in the genesis of an ectopic pregnancy.

Since the gestational sac is not present in the early stages of pregnancy, a great number of heterotopic pregnancies are diagnosed late, due to the fact that the pregnant woman may be asymptomatic or without specific symptoms such as diffuse abdominal pain and vaginal bleeding [2, 3]. Approximately 70% to 75% of the heterotopic pregnancies are discovered between the 5^th^ and 8^th^ weeks of gestation, and in only 10% of the cases, they are diagnosed past 10^th^ week of gestation [4–7, 10]. In 60% to 75%, the ectopic pregnancy is discovered during an emergency situation right before the surgery which is required due to rupture and subsequent intra-abdominal bleeding hemoperitoneum or even hypovolemic shock and is found via ultrasound or computerized tomography [4, 5, 7, 11]. However, obstetricians should always perform examination for ectopic pregnancy, abortion, and ovarian torsion [12]. B-human chorionic gonadotropin (beta-hCG) is of no value in order to differentiate cases of heterotopic pregnancies, since the pathologic hormone levels of the extrauterine pregnancy are masked by the beta-hCG produced by the intrauterine pregnancy [2, 5].

Transvaginal ultrasound represents the most useful diagnostic tool for the diagnosis of a heterotopic pregnancy, however with a sensitivity that remains low and varies between 26% and 92%, resulting in the diagnosis of about 26-41% of such cases [2, 7, 12, 13]. Moreover, multiple ultrasound scans might be required in order for the diagnosis to be undoubtedly established while all physicians should investigate the uterus and annexes as part of the first trimester ultrasound. The reason for this is the fact that although an intrauterine pregnancy does not exclude a synchronous ectopic gestation, it still remains highly infrequent; thus, the clinician should be very careful in order to subject the patient in a surgical treatment that could jeopardize a viable desired pregnancy.

Since the rupture of a tubal gestation has been reported to be as high as 20%, leading in an emergency operation, the management of a heterotopic pregnancy highly depends on several factors, which, among others, are the week of gestation, the site of the ectopic pregnancy, the experience of the physicians, and the desire of the patient to preserve the intrauterine pregnancy [6, 9, 13, 14]. In order to maintain the intrauterine pregnancy, surgical excision of the ectopic gestation through either laparoscopy or laparotomy should be performed right after diagnosis [2, 3]. The most common surgeries performed for the treatment of tubal heterotopic pregnancies are salpingectomy, salpingotomy, or even salpingo-oophorectomy [9]. In our case, after thorough consultation of the patient, due to the hemoperitoneum found in the sonographic evaluation and her hemodynamic stability, surgery through a minimally invasive route (laparoscopy) was employed. Based on the international literature, the viability of the intrauterine pregnancy resulting in the delivery of a live newborn after surgical treatment of the ectopic pregnancy can reach up to 70% [7, 10]. Conversely, the abortion rate of the intrauterine pregnancy after surgery ranges from 14% to 40%, while the preterm delivery rate has been reported to be 10% [13, 15].

## 4. Conclusions

The coexistence of an ectopic pregnancy should not automatically be ruled out in cases of intrauterine pregnancies, especially in women with a history of Assisted Reproduction Technologies. Obstetricians should always perform a meticulous ultrasound scan and suspect a tubal pregnancy, in all women with abnormal serum beta-hCG, adnexal abnormalities, or clinical symptoms. The early diagnosis and treatment of an ectopic pregnancy can be proven critical not only for maternal morbidity and mortality but also for the progression of an uncomplicated intrauterine pregnancy.
